# Effects of omega-3 fatty acid nutrition on mortality in septic patients: a meta-analysis of randomized controlled trials

**DOI:** 10.1186/s12871-016-0200-7

**Published:** 2016-07-18

**Authors:** Wei Tao, Ping-Song Li, Zhou Shen, Yu-Sheng Shu, Sen Liu

**Affiliations:** 1 Department of Burns and Plastic Surgery, Subei People’s Hospital of Jiangsu province, Jiangsu, 225001 People’s Republic of China; 2 Department of Outpatient, Subei People’s Hospital of Jiangsu province, Jiangsu, 225001 People’s Republic of China; 3 Department of Thoracic Surgery, Subei People’s Hospital of Jiangsu province, Jiangsu, 225001 People’s Republic of China

**Keywords:** Omega-3 fatty acids, Sepsis, Mortality

## Abstract

**Background:**

A previous systematic review and meta-analysis reported that omega-3 fatty acids nutrition may reduce mortality in septic patients. As new randomized controlled trials began to accumulate, we conducted an update.

**Methods:**

A PubMed database was searched through Feb 2016, and randomized controlled trials comparing omega-3 fatty acids with control were selected by two reviewers independently.

**Results:**

Eleven trials randomly assigning 808 patients were included in the present study. Using a fixed effects model, we found no significant effect of omega-3 fatty acids on overall mortality (risk ratio 0.84; 95 % confidence interval (CI): 0.67 to 1.05, *P* = 0.12), or infectious complications (risk ratio 0.95; 95 % CI: 0.72 to 1.25, *P* = 0.70). However, the duration of mechanical ventilation was markedly reduced by omega-3 fatty acids (weighted mean differences (WMD) = −3.82; 95 % CI: −4.61 to −3.04; *P* < 0.00001). A significant heterogeneity was found when the duration of hospital (*I* ^2^ = 93 %; WMD = −2.82; 95 % CI: −9.88 to 4.23, *P* = 0.43), or intensive care stay (*I* ^2^ = 87 %; WMD = −2.70; 95 % CI: −6.40 to 1.00, *P* = 0.15) were investigated.

**Conclusions:**

Omega-3 fatty acids confer no mortality benefit but are associated with a reduction in mechanical ventilation duration in septic patients. However, low sample size and heterogeneity of the cohorts included in this analysis limits the generalizability of our findings.

**Electronic supplementary material:**

The online version of this article (doi:10.1186/s12871-016-0200-7) contains supplementary material, which is available to authorized users.

## Background

Sepsis is a life threatening systemic inflammatory syndrome that is triggered by infection, and it is a major cause of death in critical ill patients such as severe burn [1, 2]. Even with dramatic advances in pharmacology as well as the technology of intensive care and life supporting, the mortality estimates in septic patients is as high as over 25 % [1]. It is suggested that when complicated with organ failure or shock, the mortality in septic patients could be further elevated to 33.2 % [3–5]. Thus, strategies that could play a benefit on survival in septic patients are appreciated.

Omega-3 fatty acids are reported to have a protective role in cardiovascular diseases [6]. And it is also recognized that omega-3 fatty acids have anti-inflammatory and immunomodulating features [7]. Recently, a number of studies have focused on the effect of omega-3 fatty acids on septic patients [8–17]. However, inconsistent findings were reported between trials. A systematic review and meta-analysis published in 2014 found mortality in septic patients could be reduced by parenteral nutrition of omega-3 fatty acids [18]. However, as the low quality of enrolled trials, the authors concluded that the effect of omega-3 fatty acids on the mortality of sepsis was uncertain [18]. Meanwhile, as new randomized controlled trials began to accumulate, we conducted an update and in the present meta-analysis, our goal was to evaluate the effect of omega-3 fatty acids nutrition on mortality, the duration of hospital, intensive care unit (ICU) stay, infectious complications, and the duration of mechanical ventilation in septic patients.

## Methods

### Search strategy

The present study was conducted according to the Preferred Reporting Items for Systematic reviews and Meta Analysis (PRISMA) of randomized controlled trials [19]. We conducted an electronic search of PubMed database up to Feb 2016 for relevant studies that tested the effect of omega-3 fatty acids enriched nutrition on septic patients. And we limited citations to prospective randomized controlled trials in adults. The following search terms were used: 1) omega-3 fatty acids, fish oil, eicosapentaenoic acid, or docosahexaenoic acid; 2) sepsis, severe sepsis, or septic shock. In order to identify other potentially eligible studies that had not been captured with our primary electronic search, the reference lists of retrieved published papers and recent reviews were also reviewed. The literature language was restricted to English.

### Study selection

Two investigators (WT and ZS) reviewed studies for inclusion, retrieved potentially relevant studies, and decided on study eligibility independently. Any differences were resolved by consensus. Agreements of trial eligibility between reviewers were assessed by using Cohen’s k [20]. As the current meta-analysis was based on trials which published previously, no ethical approval or patient consent was required.

### Inclusion criteria

Studies were considered eligible if the following criteria were present: 1) the study design was a randomized controlled trial in adults (≥18 years of age); 2) subjects were patients with sepsis, severe sepsis, or septic shock [21]; 3) the study compared omega-3 fatty acids enriched nutrition (administrated via either parenteral or enteral route) with control (septic patients treated with an omega-3 fatty acids deficiency nutrition) ; 4) the mortality (at 28-days or, if not available, mortality at any time was recorded), the length of ICU stay, the duration of hospital, infectious complications, or the duration of mechanical ventilation was reported.

### Exclusion criteria

The exclusion criterion was: using healthy people as control, administration strategies were different between study and control group, the article was a review, editorial, children’s research, animal study, letter or other type of publication not based on original research, or the study did not include extractable outcomes.

### Data extraction and quality assessment

The following items from the each study were extracted into standardized data abstraction forms by two reviewers (WT and ZS) independently: first author’s last name, publication year, sample size, country of origin, the composition of nutrition, administration strategies, and secondary organ dysfunction. The primary outcome was mortality. The secondary outcomes were the length of ICU stay, the duration of hospital stay, infectious complications, and the duration of mechanical ventilation. The methodological quality of each trial was evaluated by two of the researchers (WT and ZS) independently, and the Jadad’s scale and Cochrane Handbook for risks of bias assessments were used [22, 23].

### Statistical analysis

RevMan software (version 5.3.5) was used in our meta-analysis. We reported binary outcomes as risk ratios (RR) and continuous outcomes as weighted mean differences (WMD). Summary effect estimates were presented with 95 % confidence interval (CI). Heterogeneity was explored for all the meta-analysis. Statistical heterogeneity was measured and quantified using the I^2^ test and the Mantel–Haenszel χ2 test. Statistical heterogeneity was predefined at *I* ^2^ > 50 % or *P* < 0.05 by Mantel–Haenszel χ2 test. We reported results from a fixed effect model if there was no statistical heterogeneity (*P* ≥ 0.05 or *I* ^2^ ≤ 50 %), or, a randomized effect model was used. Publication bias was assessed for all analysis after visual inspection of funnel plots. The Cohen’s k was used to assess the agreements between reviewers for trial eligibility and methodological quality assessment [20].

## Results

### Search results

**Fig. 1:**
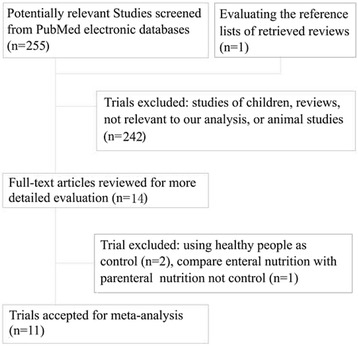
Flowchart shows literature evaluated at each stage of the meta-analysis

256 potentially relevant articles were identified after an electronic searching from among 255 listed in the PubMed database, and one article after evaluating the reference lists of retrieved reviews. Most of the 256 articles were excluded for reasons such as studies of children, reviews, not relevant to our meta-analysis, or animal studies after screening titles and abstracts. Then, fourteen relevant trials were yielded [8–17, 24–27]. After a full-text assessment, three trials [24, 25, 27] were excluded for reasons such as using healthy people as control [24, 25], comparing enteral with parenteral nutrition of omega-3 fatty acids not control (septic patients treated with an omega-3 fatty acids deficiency nutrition) [27] (Fig. 1). Finally, we included eleven randomized controlled trials [8–17, 26] in the present meta-analysis which were completely agreed by two reviewers (*k* = 1). We excluded 8 trials that included in a previous meta-analysis [18] based on our inclusion and exclusion criteria (5 published in Chinese language, two using healthy people as control, one was published as abstracts). Our study included four trials [8, 12, 13, 17] investigated the effect of enteral omega-3 fatty acids nutrition on septic patients. And three new randomized controlled trials [9–11] investigate parenteral nutrition were included in the present meta-analysis. Figure 1 shows a flowchart of study selection at different stages of the meta-analysis.

### Characteristics and the quality of studies

The included eleven trials were published from 2003 to 2015 and enrolled an aggregate of 808 patients, including 405 patients in the study group and 403 in the control group. We did not have access to primary data and therefore were reliant on the case definitions of sepsis used by the authors of the individual studies. The sizes of studies ranged from 25 to 198. Table 1 presents the characteristics of the 11 randomized controlled trials which were conducted in Europe, Brazil, or Asia. Most of the randomized controlled trials were single center, except two trials were multicentre studies [12, 13]. Additional file 1: Table S1 shows detailed information about the quality assessment of the included studies, including the Jadad score and the results of allocation concealment.

**Table 1:** Details of included trials

Author	Population	Route of Administration	Details of administration		Mortality
			Study	Control	
Shirai, 2015	1 center, 46 patients, sepsis-induced ARDS	EN	Oxepa	Ensure Liquid	60 days
Hall, 2015	1 center, 60 patients, sepsis	PN	Omegaven	standard medical care	28 days
Burkhart, 2014	1 center, 50 patients, sepsis	PN	Omegaven	standard treatment	Hospital
Gultekin, 2014	1 center, 58 patients, severe sepsis or septic shock	PN	Omegaven + ClinOleic	ClinOleic	Unspecified
Pontes-Arruda, 2011	5 centers, 115 patients, early stages of sepsis	EN	Oxepa	Ensure Plus HN	28 days
Grau-Carmona, 2011	11 centers, 198 patients, septic patients with ALI or ARDS	EN	Oxepa	Ensure Plus HN	Unspecified
Khor, 2011	1 center, 28 patients, severe sepsis	PN	Omegaven	normal saline	normal saline
Barbosa, 2010	1 center, 25 patients, sepsis or SIRS	PN	Lipolus	NuTRIflex Lipid Special	28 days
Friesecke, 2008	1 center, 166 patients, critically ill patients (115 SIRS, 51 non-SIRS)	PN	Omegaven + Lipofundin	Lipofundin	28 days
Pontes-Arruda, 2006	1 center, 165 patients, severe sepsis or septic shock	EN	EPA-GLA enriched diet	Control Diet	28 days
Grecu, 2003	1 center, 54 (15 in ICU) patients, abdominal sepsis	PN	Omegaven + LCT	LCT	ICU

*ARDS* acute respiratory distress syndrome, *ALI* acute lung injury, *SIRS* Systemic inflammatory response syndrome, *EN* enteral nutrition, *PN* parenteral nutrition, *LCT* Long-chain triglycerides, *EPA* eicosapentaenoic acid, *GLA* gamma-linolenic acid, Ensure Liquid, Ensure Plus HN, omega-3 fatty acids deficiency nutrition for EN; Lipofundin, NuTRIflex Lipid Special, ClinOleic, omega-3 fatty acids deficiency nutrition for PN; Oxepa, omega-3 fatty acids enriched nutrition for EN; Omegaven, omega-3 fatty acids enriched nutrition for PN

### Effect of omega-3 fatty acids on mortality

**Fig. 2:**
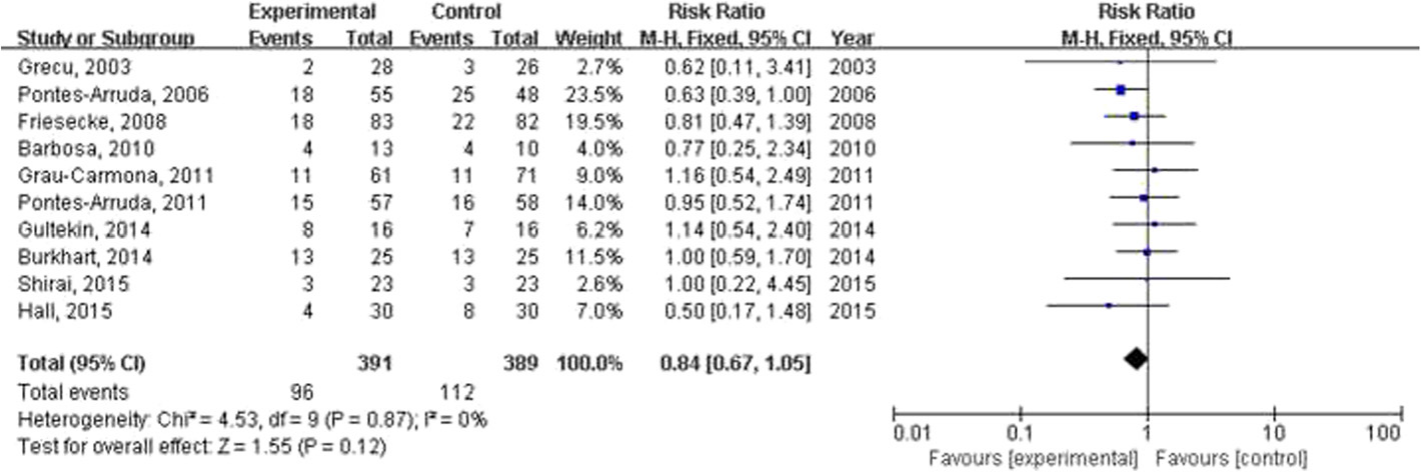
Forest plots show the effect of omega-3 fatty acids on mortality in septic patients

**Fig. 3:**
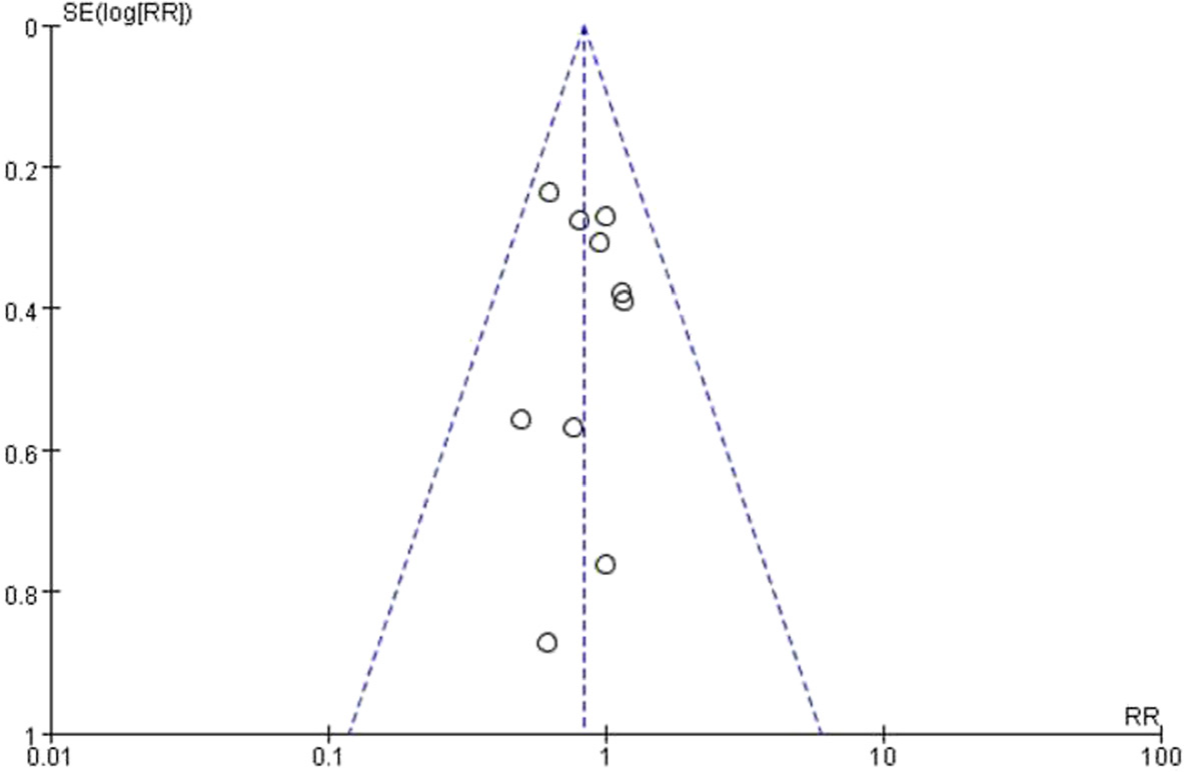
Funnel plots analysis of mortality in trials of omega-3 fatty acids nutrition in septic patients. Each point represents one trial

Mortality was reported in ten studies, we combined these trials in the mortality analyses. A fixed effect model was used, as no heterogeneity was observed across the trials (*I* ^2^ = 0 %; Fig. 2). Our meta-analysis showed that omega-3 fatty acids did not reduce mortality (risk ratio 0.84; 95 % CI: 0.67 to 1.05, *P* = 0.12; Fig. 2). However, a publication bias may exist in the meta-analysis that was focused on the mortality by the funnel plot analysis (Fig. 3).

### Effect of omega-3 fatty acids on infectious complications or the duration of mechanical ventilation

**Fig. 4:**
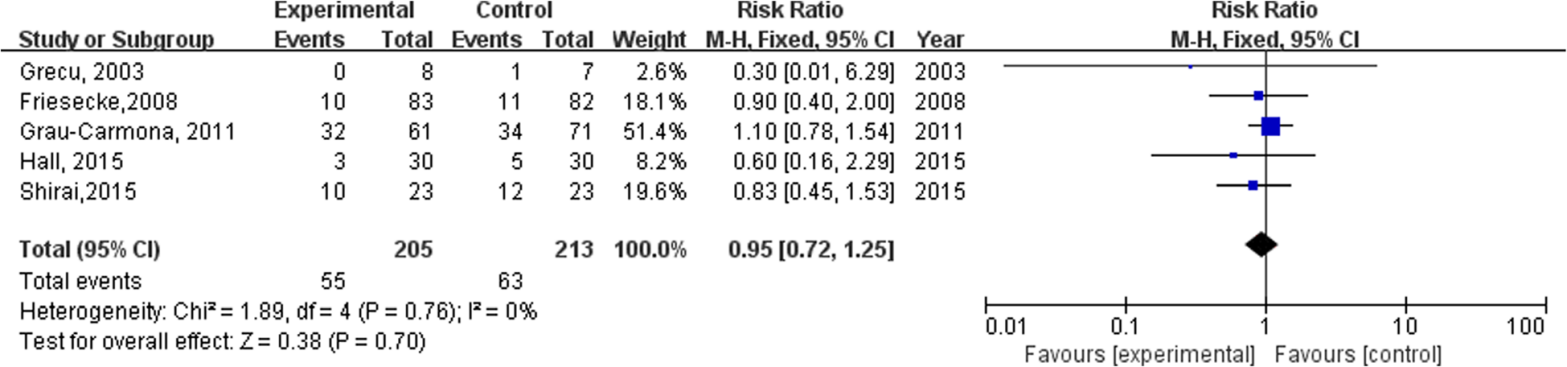
Forest plots show the effect of omega-3 fatty acids on infectious complications in septic patients

Infectious complications were reported in five studies (Additional file 2: Table S2), we combined these trials in the meta-analysis. Our meta-analysis showed that omega-3 fatty acids did not affect infectious complications (risk ratio 0.95; 95 % CI: 0.72 to 1.25, *P* = 0.70; Fig. 4).

**Fig. 5:**
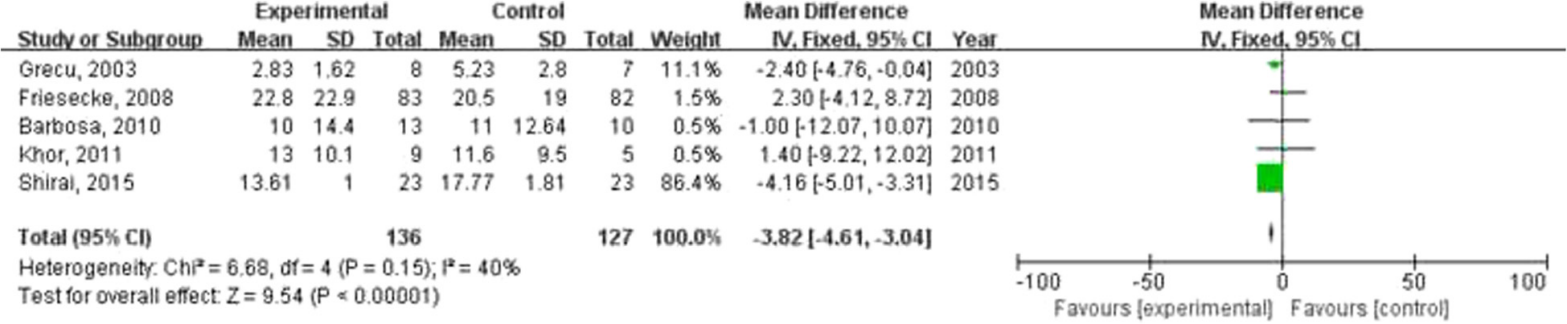
Forest plots show the effect of omega-3 fatty acids on the duration of mechanical ventilation in septic patients

The duration of mechanical ventilation was reported in seven studies, but the data from five of them could be combined in the meta-analysis (Additional file 2: Table S2). Our meta-analysis showed that omega-3 fatty acids can reduce the duration of mechanical ventilation (WMD = −3.82; 95 % CI: −4.61 to −3.04; *P* < 0.00001; Fig. 5). However, a heterogeneity was found across the trials (*I* ^2^ = 40 %; Fig. 5). Sensitivity analyses suggested that no benefit on the duration of mechanical ventilation was observed among the remaining studies after excluded one study [8] (*I* ^2^ = 0 %; Additional file 3: Figure 5S). Thus, this result should be interpreted with caution.

### Effect of omega-3 fatty acids on the length of ICU or hospital stay

Although 9 trials presented the length of ICU stay, the data from six of them could be analyzed in the meta-analysis (Additional file 4: Table S3). A marked heterogeneity was observed across the trial (*I* ^2^ = 87 %; Additional file 5: Figure 1S1). Additional file 5: Figure 1S1 shows the result from a randomized effect model that investigates the effect of omega-3 fatty acids on the length of ICU stay. According to our results, omega-3 fatty acids did not affect the length of ICU stay (WMD = −2.70; 95 % CI: −6.40 to 1.00, *P* = 0.15; Additional file 5: Figure 1S1). Sensitivity analyses were conducted to explore potential sources of heterogeneity. A mild heterogeneity was observed among the remaining studies (*I* ^2^ = 33 %; Additional file 6: Figure 1S2), after exclusion of two trial [8, 26]. But the exclusion of the trial [8, 26] did not materially alter the effect of omega-3 fatty acids on the length of ICU stay (Additional file 6: Figure 1S2).

We combined four trials in the length of hospital stay analysis. Our meta-analysis showed that omega-3 fatty acids did not affect the length of hospital stay (WMD = −2.82; 95 % CI: −9.88 to 4.23, *P* = 0.43; Additional file 7: Figure 2S1). A significantly heterogeneity was observed across the trial (*I* ^2^ = 93 %; Additional file 7: Figure 2S1). Our sensitivity analyses suggested that after exclusion of one trial [26], no heterogeneity was observed among the remaining studies (*I* ^2^ = 0 %; Additional file 8: Figure 2S2). But the exclusion of the trial [26] did not materially alter the effect of omega-3 fatty acids on the length of hospital stay (WMD =0.96; 95 % CI: −1.55 to 3.48, *P* = 0.45; Additional file 8: Figure 2S2).

### Subgroup analysis

Subgroup analysis was performed to investigate that whether the effect of omega-3 fatty acids enriched nutrition on sepsis was influenced by its dose and composition, or a different administration route (either parenteral or enteral pathway). Our meta-analysis indicated that neither parenteral (risk ratio 0.84; 95 % CI: 0.61 to 1.14, *P* = 0.26; Additional file 9: Figure 3S1; included 6 trials) nor enteral (risk ratio 0.84; 95 % CI: 0.61 to 1.16, *P* = 0.29; Additional file 10: Figure 3S2; included 4 studies) omega-3 fatty acids nutrition influenced mortality in septic patients.

During the 11 trials included in the present meta-analysis, six trials investigated Omegaven (a commercially available omega-3 fatty acids enriched parenteral nutrition), however, only five of them reported mortality (risk ratio 0.84; 95 % CI: 0.61 to 1.16, *P* = 0.30; Additional file 11: Figure 4S1); Oxepa (a commercially available omega-3 fatty acids enriched enteral nutrition) was studied in three trials (risk ratio 1.03; 95 % CI: 0.66 to 1.62, *P* = 0.89; Additional file 12: Figure 4S2), one studies investigated self-made omega-3 fatty acids enriched enteral nutrition (risk ratio 0.63; 95 % CI: 0.39 to 1.00; Fig. 2) as well as one trial investigated Lipolus (a commercially available omega-3 fatty acids enriched parenteral nutrition, risk ratio 0.77; 95 % CI: 0.25 to 2.34; Fig. 2). Our meta-analysis suggested that none of the four type of omega-3 fatty acids enriched nutrition has a benefit on mortality.

## Discussion

The primary outcome of our meta-analysis suggested that omega-3 fatty acids nutrition has no benefit on reduction of mortality in septic patient. The secondary outcomes suggested that omega-3 fatty acids nutrition did not increase the infectious complications. Nevertheless, omega-3 fatty acids nutrition failed to show a beneficial effect on the length of hospital stay, or intensive care stay. However, the duration of mechanical ventilation was markedly reduced by omega-3 fatty acids.

A previous systematic review and meta-analysis of parenteral omega-3 fatty acids for sepsis found a beneficial effect on mortality (risk ratio 0.77; 95 % CI: 0.59 to 0.99, *P* = 0.04; 12 trials, 721 patients). Our study is not consistent with this work. The difference of enrolled trials may attribute to the inconsistent result between our study and the previous meta-analysis. In the present meta-analysis, we also investigated that whether the effect of omega-3 fatty acids on sepsis was different when it was administrated via parenteral or enteral pathway, and whether the dose and composition of omega-3 fatty acids nutrition could influence its effect.

Secondary organ dysfunction is a key factor that triggered death in septic patients [1, 3–5]. It has been found that omega-3 fatty acids nutrition may prevent new organ dysfunction in three randomized controlled trials with 278 cases of sepsis [9, 12, 17] (two trials investigated mild or early sepsis [9, 12], one study with severe sepsis or septic shock [17]). Thus, it seems that the omega-3 fatty acids may play a benefit on sepsis through inhibit new organ dysfunction. SOFA scores, an indicator of secondary organ dysfunction, were reported in three trials [8, 9, 13]. As it was unable to obtain explicit data on SOFA scores that could be combined in the meta-analysis, we did not analyze the effect of omega-3 fatty acids on SOFA scores. It seems that omega-3 fatty acids may have potential benefit on new organ dysfunction; we suggested that a further study focus on the effect of omega-3 fatty acids on sepsis should investigate secondary organ dysfunction. Mechanical ventilation is a lifesaving strategy for critical ill patients. However, its adverse effects such as ventilation-induced lung injury, and ventilator associated pneumonia are life threatening. Our meta-analysis included 5 trials (Fig. 5) suggested that omega-3 fatty acids can reduce the duration of mechanical ventilation in septic patient. This result is not consistent with a previous meta-analysis [18] which included two studies [15, 16]. Nevertheless, heterogeneity was found across the trials. Thus, the result should be interpreted with caution.

The potential mechanism that could possibly explain why omega-3 fatty acids may not be beneficial in septic patients was as follows: Firstly, sepsis is a severe disease with a high mortality [1, 3–5]. A number of factors such as a history of severe diseases, or divergence of pathogen may influence the clinic outcome. Septic patients often complicated with organ dysfunction or shock which may further elevate mortality. The included trials in our meta-analysis suggested that a number of patients diagnosed as sepsis were complicated organ dysfunction or shock. Acute respiratory distress syndrome, an inflammation disease with mortality over 40 %, is a common secondary organ dysfunction in septic patients [1, 3–5, 28]. A previous meta-analysis focused on the effect of omega-3 fatty acids on acute respiratory distress syndrome (ARDS) reported that it has no effect on reduction of 28-day mortality [29]. The failure on the improvement of complications by omega-3 fatty acids seems to have limited impact on septic patients’ survival. Secondly, the rational of omega-3 fatty acids nutrition as an adjunctive therapy for sepsis is partly based on its anti-inflammatory and immunomodulating features. However, the mild anti-inflammatory effect of omega-3 fatty acids might not lead to improve in outcome. It has been reported that even a delay of antibiotics treatment may cause a significant increased mortality in patients with sepsis [30].

A previous meta-analysis [29] suggested that a high fat formula used in control group which may contribute to an increased mortality could be the reason that a reduced mortality was observed in study group. It is suggested that the high fat formula may have pro-inflammatory features, and resulting in a worsened clinical outcome [31]. It seems that clinical outcomes could be influenced by the dose and composition of nutrition. As the dose and composition of omega-3 fatty acids nutrition agent were divergent in studies, we are interested in that whether the effect of omega-3 fatty acids on sepsis may be affected by the difference of dose and composition. Therefore, we examined trials testing different type of omega-3 fatty acids agent separately. Our subgroup meta-analysis indicated that the effect of omega-3 fatty acids on mortality in septic patients was not influenced by its dose and composition.

We are also interested in that whether the difference between parenteral and enteral administration may influence the effect of omega-3 fatty acids on septic patients. In a previous meta-analysis [32] included three trials that reported a protective effect of omega-3 fatty acids on ARDS, the administration strategy was enteral nutrition. Our subgroup meta-analysis suggested that the mortality in septic patients was not influenced by omega-3 fatty acids that administrated through either parenteral or enteral pathway. This result is consistent with a previous meta-analysis that focuses on the effect of omega-3 fatty acids on critically ill patients [33]. These results suggested that the administration strategy may not be the key factor that influenced the effect of omega-3 fatty acids on sepsis or other critically ill patients.

There are some limitations in our meta-analysis. One potential limitation of the present meta-analysis was the asymmetric funnel plot that suggested publication bias may exist. It is possibly that the present meta-analysis did not include all of the trials, as we restricted the literature language to English, and other database except the PubMed was not adopted. Meanwhile, the small sample size may also have been a limitation, which may have lowered the statistical power. Furthermore, a marked heterogeneity was detected when the length of ICU or hospital stay was analyzed.

In brief, although our meta-analysis suggested that omega-3 fatty acids failed to reduce overall mortality in septic patients, a tendency toward a reduction in mortality should not be neglected (risk ratio 0.84; 95 % CI: 0.67 to 1.05, *P* = 0.12). Thus, future large scale, multicentre investigation is still required to draw a conclusion.

## Conclusions

Omega-3 fatty acids confer no mortality benefit but are associated with a reduction in mechanical ventilation duration in septic patients. However, low sample size and heterogeneity of the cohorts included in this analysis limits the generalizability of our findings.

## Abbreviations

ARDS, acute respiratory distress syndrome; CI, confidence interval; ICU, intensive care unit; PRISMA, The Preferred Reporting Items for Systematic reviews and Meta Analysis; RR, risk ratios; SOFA, sequential organ failure assessment; WMD, weighted mean differences

## Additional files

Additional file 1: Table S1. Jadad scores and methodology quality. (DOC 12 kb)

Additional file 2: Table S2. Details of omega-3 fatty acids nutrition on mechanical ventilation days or infectious complications in septic patients. (DOC 14 kb)

Additional file 3: Figure 5S. Sensitivity analyses the effect of omega-3 fatty acids on the duration of mechanical ventilation in septic patients. (TIF 22 kb)

Additional file 4: Table S3. Details of Omega-3 fatty acids nutrition on new organ dysfunction, or the length of ICU or hospital stay. (DOC 18 kb)

Additional file 5: Figure 1S1. Forest plots show the effect of omega-3 fatty acids on the length of ICU stay in septic patients. (TIF 25 kb)

Additional file 6: Figure 1S2. Sensitivity analyses the effect of omega-3 fatty acids on the length of ICU stay in septic patients. (TIF 22 kb)

Additional file 7: Figure 2S1. Forest plots show the effect of omega-3 fatty acids on the length of hospita stay in septic patients. (TIF 14 kb)

Additional file 8: Figure 2S2. Sensitivity analyses of effect of omega-3 fatty acids on the length of hospital stay in septic patients. (TIF 12 kb)

Additional file 9: Figure 3S1. Forest plots show the effect of parenteral omega-3 fatty acids nutrition on mortality in septic patients. (TIF 15 kb)

Additional file 10: Figure 3S2. Forest plots show the effect of enteral omega-3 fatty acids nutrition on mortality in septic patients. (TIF 14 kb)

Additional file 11: Figure 4S1. Forest plots show the effect of omegaven on mortality in septic patients. (TIF 15 kb)

Additional file 12: Figure 4S2. Forest plots show the effect of Oxepa on mortality in septic patients. (TIF 13 kb)
