# Supplementary material for: Effects of omega-3 fatty acid nutrition on mortality in septic patients: a meta-analysis of randomized controlled trials
Source: BMC Anesthesiol. 2016 Jul 18;16:39. doi: 10.1186/s12871-016-0200-7 (PMC4950703; doi:10.1186/s12871-016-0200-7)
Supplement: Supplementary file 1 — Jadad scores and methodology quality. (DOC 12 kb) [file 12871_2016_200_MOESM1_ESM.doc]

**Table S1. Jadad scores and Allocation concealment.**

**Author Jadad scores Allocation concealment**

Shirai, 2015 2 not used

Hall, 2015 3 not used

Burkhart, 2014 3 not used

Gultekin, 2014 3 not used

Pontes-Arruda, 2011 5 adequate

Grau-Carmona, 2011 3 not used

Khor, 2011 3 adequate

Barbosa, 2010 3 not used

Friesecke, 2008 4 adequate

Pontes-Arruda, 2006 4 adequate

Grecu, 2003 4 adequate
