# Supplementary material for: Effects of omega-3 fatty acid nutrition on mortality in septic patients: a meta-analysis of randomized controlled trials
Source: BMC Anesthesiol. 2016 Jul 18;16:39. doi: 10.1186/s12871-016-0200-7 (PMC4950703; doi:10.1186/s12871-016-0200-7)
Supplement: Supplementary file 2 — Details of omega-3 fatty acids nutrition on mechanical ventilation days or infectious complications in septic patients. (DOC 14 kb) [file 12871_2016_200_MOESM2_ESM.doc]

**Table S2. Details of omega-3 fatty acids nutrition on mechanical ventilation days or infectious complications in septic patients.**

**Author** **Mechanical ventilation days Infectious complications**

**Study Control Study Control**

Shirai, 2015 13.61±1.00 17.77±1.81 10(23) 12(23)

Hall, 2015 not report not report 3(30) 5(30)

Burkhart, 2014 not report not report not report not report

Gultekin, 2014 not report not report not report not report

Pontes-Arruda, 2011 7 15 not report not report

Grau-Carmona, 2011 10 9 32(61) 34(71)

Khor, 2011 13.0±10.1 11.6±9.5 not report not report

Barbosa, 2010 10±14.4 11±12.64 not report not report

Friesecke, 2008 22.8±22.9 20.5±19.0 10(83) 11(82)

Pontes-Arruda, 2006 not report not report not report not report

Grecu, 2003 2.83±1.62 5.23±2.80 0(8) 1(7)
