# Supplementary figures and images for: Effects of omega-3 fatty acid nutrition on mortality in septic patients: a meta-analysis of randomized controlled trials
Source: BMC Anesthesiol. 2016 Jul 18;16:39. doi: 10.1186/s12871-016-0200-7 (PMC4950703; doi:10.1186/s12871-016-0200-7)

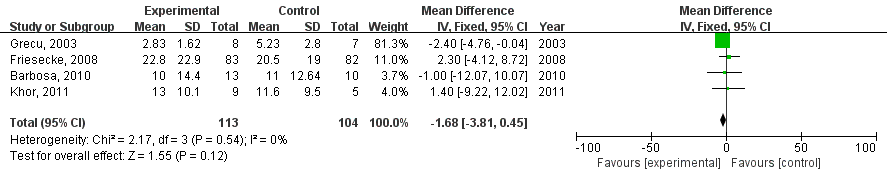

Supplement: Supplementary file 3 — Sensitivity analyses the effect of omega-3 fatty acids on the duration of mechanical ventilation in septic patients. (TIF 22 kb) [file 12871_2016_200_MOESM3_ESM.tif]

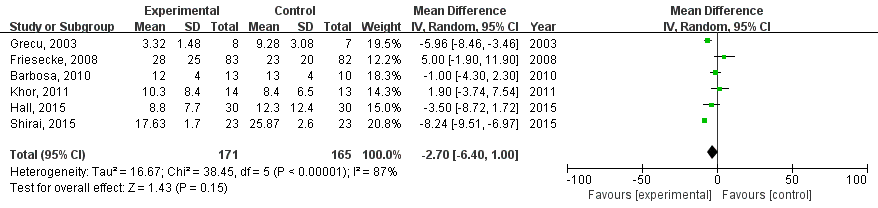

Supplement: Supplementary file 5 — Forest plots show the effect of omega-3 fatty acids on the length of ICU stay in septic patients. (TIF 25 kb) [file 12871_2016_200_MOESM5_ESM.tif]

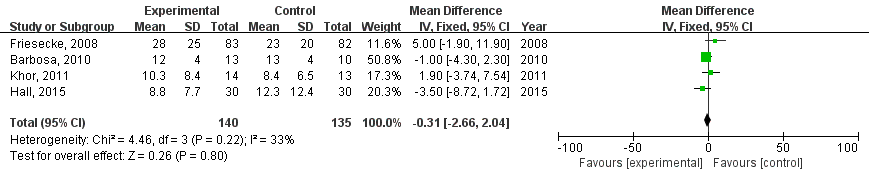

Supplement: Supplementary file 6 — Sensitivity analyses the effect of omega-3 fatty acids on the length of ICU stay in septic patients. (TIF 22 kb) [file 12871_2016_200_MOESM6_ESM.tif]

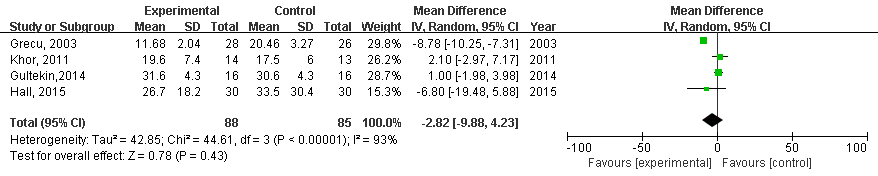

Supplement: Supplementary file 7 — Forest plots show the effect of omega-3 fatty acids on the length of hospita stay in septic patients. (TIF 14 kb) [file 12871_2016_200_MOESM7_ESM.tif]

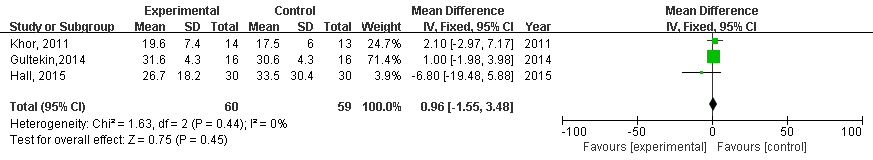

Supplement: Supplementary file 8 — Sensitivity analyses of effect of omega-3 fatty acids on the length of hospital stay in septic patients. (TIF 12 kb) [file 12871_2016_200_MOESM8_ESM.tif]

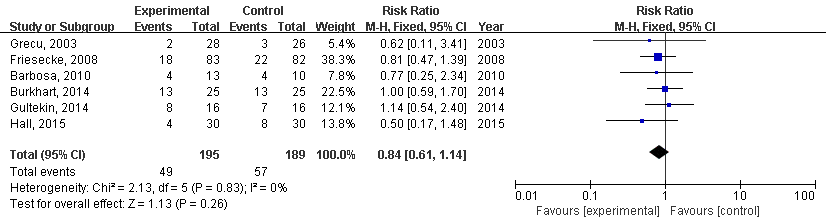

Supplement: Supplementary file 9 — Forest plots show the effect of parenteral omega-3 fatty acids nutrition on mortality in septic patients. (TIF 15 kb) [file 12871_2016_200_MOESM9_ESM.tif]

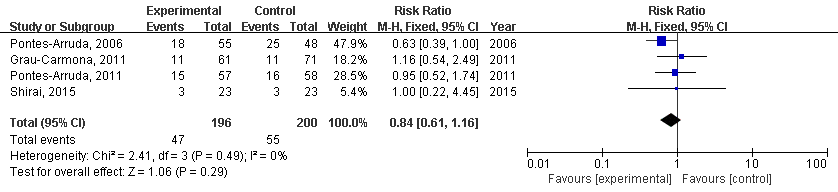

Supplement: Supplementary file 10 — Forest plots show the effect of enteral omega-3 fatty acids nutrition on mortality in septic patients. (TIF 14 kb) [file 12871_2016_200_MOESM10_ESM.tif]

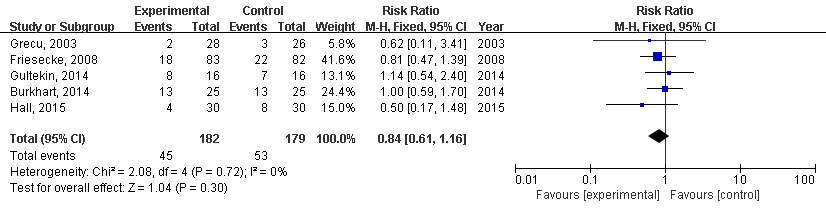

Supplement: Supplementary file 11 — Forest plots show the effect of omegaven on mortality in septic patients. (TIF 15 kb) [file 12871_2016_200_MOESM11_ESM.tif]

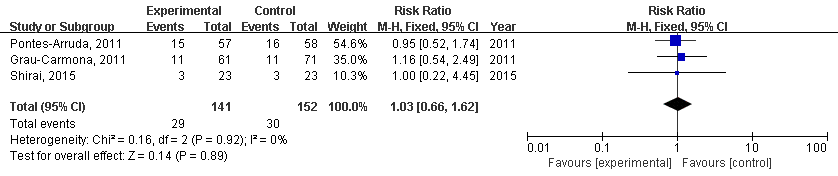

Supplement: Supplementary file 12 — Forest plots show the effect of Oxepa on mortality in septic patients. (TIF 13 kb) [file 12871_2016_200_MOESM12_ESM.tif]
