# Supplementary material for: Effects of omega-3 fatty acid nutrition on mortality in septic patients: a meta-analysis of randomized controlled trials
Source: BMC Anesthesiol. 2016 Jul 18;16:39. doi: 10.1186/s12871-016-0200-7 (PMC4950703; doi:10.1186/s12871-016-0200-7)
Supplement: Supplementary file 4 — Details of Omega-3 fatty acids nutrition on new organ dysfunction, or the length of ICU or hospital stay. (DOC 18 kb) [file 12871_2016_200_MOESM4_ESM.doc]

**Table S3. Details of omega-3 fatty acids nutrition on new organ dysfunction, or the length of ICU or hospital stay.**

**Author** **Length of stay New organ dysfunction**

**Study Control**

Shirai, 2015 ICU 17.63±1.70 ICU 25.87±2.6 SOFA scores were not significantly different between study and control groups.

Hospital not report

Hall, 2015 ICU 8.8±7.7 ICU 12.3±12.4 Δ-SOFA 2.2 ± 2.2 (control) vs. 1.0 ± 1.5 (study), P = 0.005

Hospital 26.7±18.2 Hospital 33.5±30.4 maximum-SOFA 10.1 ± 4.2 (control) vs. 8.1 ± 3.2 (study), P =0.041

Burkhart, 2014 ICU 5 (3 to 22) ^#^ ICU 6 (2 to 33) ^#^ not report

Hospital not report

Gultekin, 2014 ICU not report not report

Hospital 31.6±4.3 Hospital 30.6±4.3

Pontes-Arruda, ICU 7 days (4 to 12) ^#^ ICU 13 days (9 to 18) ^#^ cardiovascular failure (36.2% (control) versus 21% (study), P = 0.0381)

2011 Hospital not report respiratory failure (39.6% (control) versus 24.6% (study), P = 0.0362)

Grau-Carmona, ICU 16 (11-25) ^#^ ICU 18 (10--30) ^#^ toward a decreased SOFA score in study group, but it was not significant.

2011 Hospital not report

Khor, 2011 ICU 10.3±8.4 ICU 8.4±6.5 not report

Hospital 19.6±7.4 Hospital 17.5±6.0

Barbosa, 2010 ICU 12±4 ICU 13±4 not report

Hospital not report

Friesecke, 2008 ICU 28±25 ICU 23±20 not report

Hospital not report

Pontes-Arruda, ICU not report new organ dysfunction (38% (study) vs. 81% (control), P<0.001)

2006 Hospital not report

Grecu, 2003 ICU 3.32±1.48 ICU 9.28±3.08 not report

Hospital 11.68±2.04 Hospital 20.46±3.27

ICU, Intensive care unit; SOFA, Sequential Organ Failure Assessment; # date could not be combined in the meta-analysis.
